# Beyond monetary value: how reward type drives cheating in a gender-judgment task

**DOI:** 10.3389/fpsyg.2024.1290793

**Published:** 2024-05-20

**Authors:** Guan-Zhao Chen, Fei-Fei Zhao, Hao-Ming Li, Yu-Wei Wu, Wen-Jing Yan

**Affiliations:** ^1^Wenzhou Seventh People’s Hospital, Wenzhou, China; ^2^School of Mental Health, Wenzhou Medical University, Wenzhou, China; ^3^Student Affairs Division, Wenzhou Business College, Wenzhou, China; ^4^Zhejiang Provincial Clinical Research Centre for Mental Illness, Affiliated Kangning Hospital, Wenzhou Medical University, Wenzhou, China

**Keywords:** cheating behavior, self-concept maintenance theory, reward type, social cognition, dishonesty

## Abstract

**Background:**

Investigating the effects of monetary incentives on dishonest behavior provides valuable insights into human integrity and ethical decision-making processes. This study is conducted through the lens of self-concept maintenance theory.

**Aim:**

The aim of this study is to examine the influence of different types of rewards (score-based vs. monetary) and their magnitude on dishonest behavior within a gender judgment task.

**Method:**

Using a quantitative experimental design, this study involved 116 participants who were randomly assigned to conditions that differed in reward type (score or money) and magnitude (10 yuan vs. 50 yuan). Dishonest behavior was assessed using a gender judgment task with mechanisms to simulate conditions conducive to planned cheating.

**Results:**

Results revealed significant differences in dishonesty rates between score and money conditions, with a higher proportion of dishonest participants observed in the score condition compared to the money condition. The timing of initial cheating was earlier in the score condition compared to the money condition. No significant differences were found in the proportion of dishonest participants, the cheating rate, or the timing of initial cheating across reward levels within either condition. The rate of cheating increased over time, suggesting a temporal dynamic in unethical decision making.

**Conclusion:**

The study demonstrates that the nature of rewards significantly influences the likelihood of dishonest behavior, with intangible score-based rewards facilitating rationalizations for dishonesty more readily than tangible financial incentives. These findings enrich the understanding of moral psychology by highlighting the complex interplay between reward types, ethical rationalization, and the dynamics of dishonest behavior.

## 1 Introduction

In behavioral psychology, the study of moral decision-making under the influence of financial incentives is a central avenue for understanding human integrity and ethical behavior. As well as exploring fundamental aspects of human nature, this area of research investigates how external factors, such as financial rewards, can influence ethical judgements and actions. The specific topic of interest within this broad area is the impact of financial rewards on dishonest behavior, particularly in situations where individuals are faced with choices that test their moral compass. The importance of this research lies in its potential to unravel the complexities of moral psychology and provide insights into how incentives shape ethical decision-making processes.

### 1.1 Monetary incentives and behavior

The influence of monetary incentives on human behavior constitutes a fundamental research domain within behavioral economics and psychology, offering rich insights into how financial rewards shape motivation, decision-making processes, and ethical conduct. At the core of this body of research is the proposition that monetary incentives can significantly motivate individuals to improve performance across a variety of tasks and settings. For instance, Shibly and Weerasinghe (2019) has demonstrated that financial rewards can enhance attention, effort, and persistence on tasks, leading to improved outcomes in both workplace settings and experimental environments. However, the relationship between monetary incentives and behavior is complex and multifaceted. The impact of financial rewards is not universally positive and varies considerably across different contexts and individual predispositions. For example, while some research highlights the efficacy of incentives in boosting performance, other studies caution against potential detrimental effects on intrinsic motivation and creativity (Bruno et al., 2017). This dichotomy underscores the nuanced interplay between extrinsic and intrinsic motivators and their collective impact on behavior.

The ethical dimension of monetary incentives further complicates their behavioral effects. Research in this area explores how financial rewards might encourage dishonesty or unethical behavior under certain conditions (Park et al., 2022). Researchers have found that individuals are more likely to engage in deceptive behaviors when doing so could result in monetary gain, suggesting that the allure of financial rewards can sometimes overpower moral considerations (Gneezy, 2005; Balasubramanian et al., 2017) when rewards are large enough (Kajackaite and Gneezy, 2017) as well as in collaborative dishonesty (Leib et al., 2021). These findings are particularly relevant in contexts where the boundaries of ethical conduct are not clearly defined, or where the perceived benefits of dishonesty outweigh the potential costs.

### 1.2 Self-concept maintenance theory and cheating behavior

Self-concept maintenance theory (Mazar et al., 2008) introduces the concept of cognitive dissonance in cheating, suggesting that individuals balance their desire for self-benefit with their need to maintain a positive self-image. This balancing act leads to a nuanced understanding of when and why people decide to cheat, highlighting the importance of internal moral standards and the justifications for unethical behavior (Shalvi et al., 2011, 2015; Guzikevits and Choshen-Hillel, 2022; Weisel and Shalvi, 2022). The “fudge factor” suggests that there is a level of cheating that can be rationalized by individuals, allowing them to benefit from dishonesty without significant harm to their self-image (Shalvi et al., 2012; Zhao et al., 2019; Qu et al., 2020). The size of the “fudge factor” varies among individuals and situations, influenced by the potential justifications for dishonest behavior. The ability to rationalize dishonest behavior plays a critical role in self-concept maintenance. Rationalizations can take many forms, such as downplaying the consequences, displacing responsibility, or dehumanizing the victims of dishonest acts. These rationalizations allow individuals to engage in dishonest behavior while minimizing the impact on their self-concept (Bandura, 1990).

Research contrasting tangible rewards (such as money) with intangible rewards (such as points or scores) suggests that tangible rewards may have a stronger influence on encouraging unethical behavior due to their material value (Vohs et al., 2006, 2008). This is in line with the concept of materialism, where the value placed on material success can overshadow ethical considerations. However, the intrinsic value associated with achieving a high score or status, despite being an extrinsic reward, can also motivate cheating, illustrating the complex interplay between different types of extrinsic motivations and their impact on behavior. Ariely and Jones (2012) expanded on the work of Mazar et al. (2008), demonstrating that the likelihood of cheating increases when people perceive a higher degree of separation from the dishonest act, such as when cheating for tokens exchangeable for money instead of directly for money. Based on self-concept maintenance theory, study also found that there was no difference between different reward levels, suggesting that people were generally insensitive to the expected external costs and benefits associated with the dishonest acts, but they were very sensitive to contextual manipulations related to the self-concept (Mazar et al., 2008). This suggests that psychological distance from the dishonest act can affect the decision-making process.

### 1.3 The present study

Despite the rich body of literature on dishonesty and the influence of rewards, several gaps remain. Notably, there is a need for more nuanced understanding of how specific reward structures—such as the difference between score-based and monetary incentives—impact the propensity of dishonest participants. Additionally, the effect of reward magnitude within these different reward types on cheating behavior is not well understood. Most studies have not directly compared these aspects in a controlled experimental design, nor have they examined the temporal dynamics of cheating behavior in relation to different types of rewards.

This study aims to fill these gaps by investigating the influence of reward type (score versus money) and magnitude (10 yuan versus 50 yuan) on dishonest behavior in a gender judgment task, an innovative experimental design that allows us to simulate conditions conducive to planned cheating. This task allow us set an unmet goal which was more likely to elicit intentional cheating in some subjects (Schweitzer et al., 2004) and allow for the detailed process of cheating to be recorded, such as when participants began cheating, how often they cheated, and whether the cheating increased (i.e., a higher rate of cheating in the latter rather than former phases) as they finished more blocks to meet the goal, in order to closely scrutinize the influence of reward on cheating behavior. We hypothesized that:

H1: More people cheat and cheat more in the score condition than in the money condition. According to self-concept maintenance theory, the degree of lying depends on the extent to which self-justifications are available; people are also more likely to lie if they can more easily justify their lies to themselves. Claiming more points, rather than more money, provides more room for justification, allowing people to interpret their dishonesty in a more self-serving way. Therefore, score not only allows people to increase their acceptable level of dishonesty, it also frees some participants from the shackles of their morality. It would also be easier for people to overcome self-concept maintenance motivation and thus engage in cheating behavior earlier in the score condition.

H2: According to Mazar et al.’s (2008) self-concept maintenance theory, no difference will be found between two reward levels.

H3: Based on a previous study by Gneezy et al. (2013), we speculate that cheating behaviors increase over time.

## 2 Method

### 2.1 Participants

A total of 116 students participated in this study. Participants were recruited via the Internet. A power analysis was conducted using G*Power 3.1.9.7 for a 2×2 factorial ANOVA with a medium effect size (*f* = 0.25), an alpha level of 0.05, and a power of 0.80. The results indicated a required total sample size of 128 participants (32 per cell). While our total sample size (*N* = 116) is slightly below this recommendation, it is still within an acceptable range. Of these, 60 students (*M*_age_ = 22.28, SD = 1.77; 30 females) were randomly allocated to one of the two conditions: either a 10 yuan (16males; 14 females) or a 50 yuan reward (14 males; 16 females). The remaining 56 students (*M*_age_ = 21.98, SD = 2.18; 29 females) were randomly assigned to money conditions: either a 10 yuan (13 males; 14 females) or a 50 yuan reward (14 males; 15 females). All participants, including those in the score condition, received a fixed monetary compensation for their participation in the study. At the time of the study, 10 yuan was approximately equivalent to 1.50 USD, and 50 yuan was approximately equivalent to 7.50 USD All participants provided written informed consent, and the study was approved by the Ethics Committee of Ludong University in accordance with the Declaration of Helsinki.

### 2.2 Stimuli

All 30 facial images from the NimStim database used in the study displayed neutral expressions. Among these 30 images, 14 featured female models, and 16 featured male models. Each image was presented twice within a single block, resulting in a total of 60 trials per block.

### 2.3 Procedure

Upon entering the lab, participants in the score conditions were seated individually at computer stations and provided with a set of noise-canceling headphones. They received the following standardized instructions on their screens: “*This study aims to assess your ability to judge the gender of the models displayed. After the presentation of facial picture, you should respond whether it is male or female. Then the correct answer appeared, if your judgment aligns with the computer’s, click ‘correct’ to earn 10 points. Conversely, click ‘wrong’ to lose 10 points. The experiment will conclude once you accumulate a total of 500 points*” (note: participants in the score condition earned points for correct responses and lost points for incorrect responses. They did not directly earn money based on their performance in the task).

Participants continued through a series of blocks until they either achieved a total of 500 points within a single block or failed to reach this criterion within five attempts. Each trial sequence commenced with a fixation cross presented for a duration of 800 to 1,200 ms, followed by the display of a facial image for 20 ms. Pre- and post-masks were each displayed for 50 ms. Feedback about the participant’s total score was then presented on-screen for 1,000 ms. The next trial was initiated after an inter-trial interval lasting between 800 and 1,200 ms.

A previous study showed that people with unmet goals were more likely to engage in unethical behavior than people attempting to do their best (Schweitzer et al., 2004). To induce participants’ cheating behaviors, based on previous study, which found that facial expressions displayed for less than 40 ms, coupled with masking, are highly challenging to recognize (Morris et al., 1998), the display of a facial image for 20 ms. Before the main study, pilot tests were conducted to verify that the task was nearly impossible to complete solely based on one’s ability to accurately identify gender.

In the money conditions, the procedure was identical to the score conditions, except that money was the unit of reward. For instance, in the 10 yuan money condition, participants saw their earnings increase or decrease by 0.2 yuan for each ‘correct’ or ‘wrong’ selection, respectively. In the 50 yuan money condition, participants saw their earnings increase or decrease by 1 yuan for each ‘correct’ or ‘wrong’ selection, respectively. Actually, the difference between score and money conditions is only labelling difference.

To make participants aware that the scoring and money was associated with moral choices and not solely dependent on the accuracy of gender identification, we incorporated a strategy inspired by Ruedy et al. (2013). Specifically, the positions of the ‘correct’ and ‘incorrect’ buttons were switched intermittently every seven trials during the first block. This tactic was intended to prompt accidental clicks, thereby drawing participants’ attention to the actual mechanics of the scoring system. The data from these initial trials were later excluded from the final analysis.

## 3 Results

For the purposes of this study, participants were categorized as “dishonest” only if they exhibited cheating behavior in more than two trials within a single block to prevent honest mistakes (Heyman et al., 2020). This threshold was set to account for accidental or unintended clicks and equates to 3% of the total data per block.

### 3.1 Proportion of dishonest participants (to test whether more people cheat in the score condition)

In the 10-point score condition, 83.33% of participants (25 out of 30) were classified as dishonest. In the 50-point score condition, this figure was 70.00% (21 out of 30). A Chi-square test revealed no significant difference between the two conditions, *χ*^2^(1, *n* = 60) = 1.49, *p* = 0.22.

**Figure 1:**
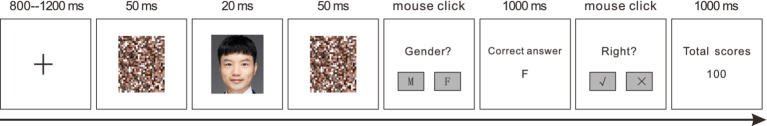
Example of a single trial in the experiment.

In the 10 yuan money condition, 59.26% of participants (16 out of 27) were classified as dishonest, while in the 50 yuan money condition, this figure was 51.72% (15out of 29). A Chi-square test indicated no significant difference between these conditions, *χ*2(1, *n* = 56) = 0.32, *p* = 0.57 (see Figure 1).

**Figure 2:**
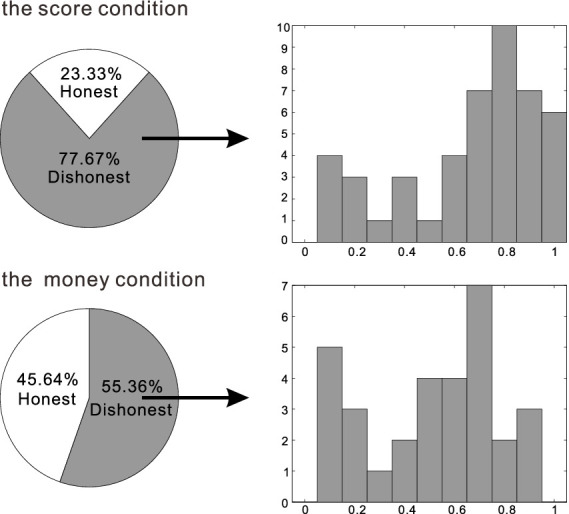
The distribution of honest and dishonest participants. The pie graph shows the proportion of honest and dishonest participants. The histogram shows the distribution of dishonest participants’ cheating rates (the highest among the blocks) in the score and money condition in detail.

To investigate the influence of reward type on cheating, we compared the proportion of dishonest participants between score and money conditions. In the score condition, 76.67% (46 out of 60) were dishonest, while in the money condition, the rate was 55.36% (31 out of 56). A Chi-square test indicated a significant difference between these conditions, *χ*2 (1, *n* = 116) = 5.89, *p* = 0.02 (see Figure 2).

### 3.2 The process of cheating behavior

Total cheating rate (to test whether people cheated more in the score condition). The cheating rate between the score (*M* = 0.46, *SE* = 0.29) and money (*M* = 0.28, *SE* = 0.24) condition was significant, *t* (75) = 2.86, *p* = 0.01 (Table 1). No significant difference was found in the total cheating rate between the two reward magnitude levels 10 yuan (*M* = 0.43, *SD* = 0.29) and 50 yuan (*M* = 0.49, *SD* = 0.29) in the score condition, *t* (44) = −0.68, *p* = 0.50. No significant difference was found in the total cheating rate between the two reward magnitude levels 10 yuan (*M* = 0.30, SD = 0.26) and 50 yuan (*M* = 0.26, SD = 0.23) in the money condition, *t* (29) = 0.49, *p* = 0.63.

**Table 1:** Description of cheating behavior.

Condition	Total cheating rate			Timing of initial cheating		Change in cheating rate			
						First block		Last block	
Score	10 yuan	0.46	0.43	1.71	1.92	0.27	0.22	0.63	0.61
	50 yuan		0.49		1.48		0.31		0.64
Money	10 yuan	0.28	0.30	2.61	2.75	0.11	0.12	0.45	0.52
	50 yuan		0.26		2.47		0.11		0.38

Timing of initial cheating behavior (to test whether people cheated earlier in the score condition). The “first cheating block” was defined as the earliest block during which a participant displayed dishonest behavior. The data is non-normally distributed; therefore the Mann–Whitney test was used. Results revealed a significant difference in the initial cheating block between the score (*M* = 1.71, SE = 1.34) and money conditions (*M* = 2.61, SE = 1.38), *z* = −2.04, *p* = 0.04 (Table 1). No significant difference was found in the timing of the initial cheating block between the two reward magnitude level 10 yuan (*M* = 1.92, SD = 1.41) and 50 yuan (*M* = 1.48, SD = 1.25) in the score condition, *z* = −1.43, *p* = 0.15. No significant difference was found in the timing of the initial cheating block between the two reward magnitude levels 10 yuan (*M* = 2.75, SD = 1.53) and 50 yuan (*M* = 2.47, SD = 1.25) in the money condition, *z* = −0.26, *p* = 0.80.

**Figure 3:**
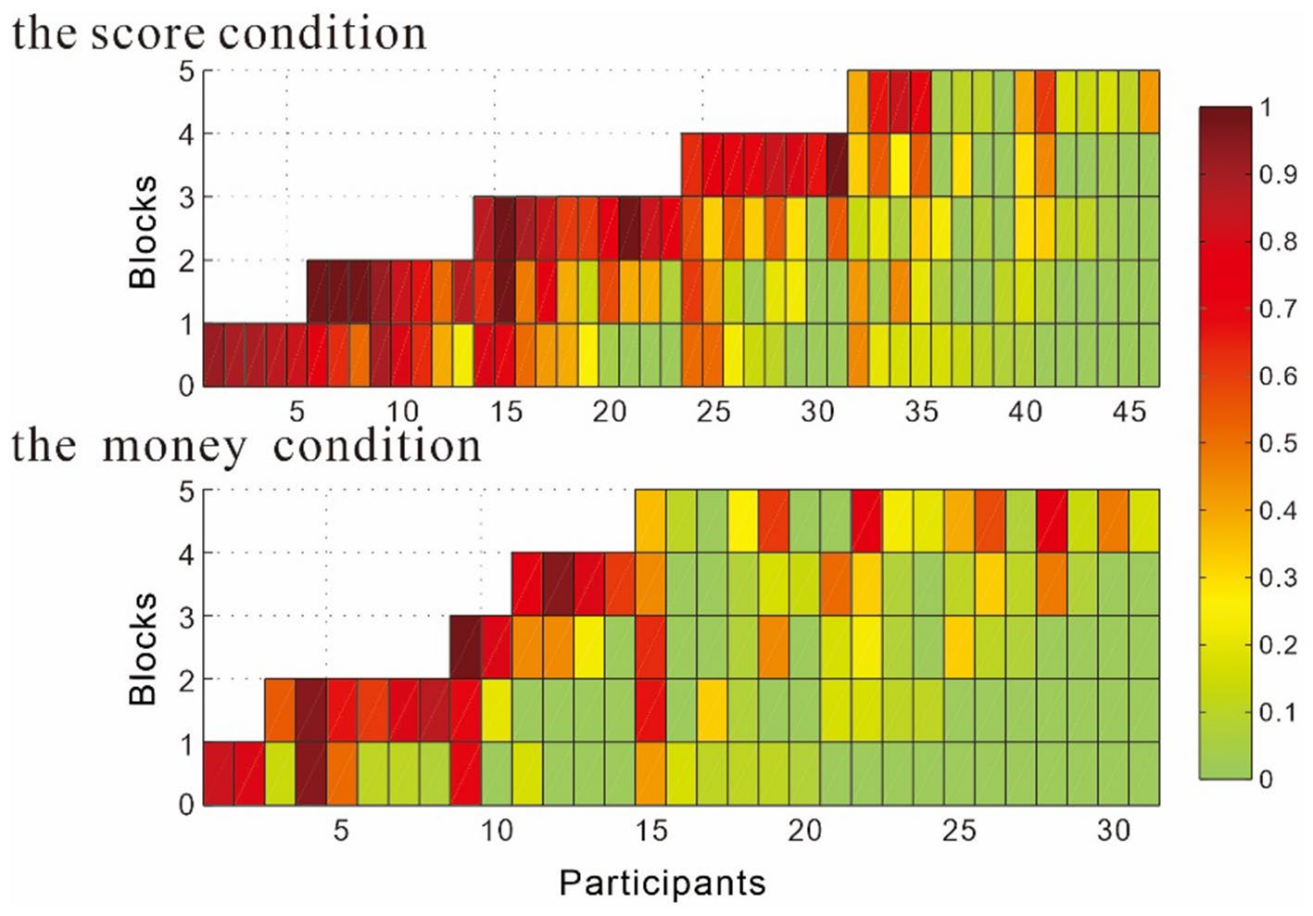
Changes in cheating rates across blocks. The x-axis indicates the index of participants, and the y-axis illustrates the index of blocks. Different colors demarcate different cheating rates. The order of presentation was sorted beginning with the subject with the least number of blocks in the score condition and money condition.

Change in cheating rate over time (to test whether cheating increased over time). To examine the evolution of cheating behavior throughout the task, a repeated-measures ANOVA was conducted. The within-subject variable was the cheating rate by block (first vs. last), and the between-subjects variable was the cheating rate by reward level (score vs. money). A total of 7 participants (5 in the score condition and 2 in the money condition) completed the task within one block and were excluded from the analysis of changes in cheating rate over time. The results showed that the cheating rate for the first block (*M* = 0.19, SD = 0.03) was significantly lower than that for the last block (*M* = 0.54, SD = 0.04), *F* (1, 68) = 92.96, *p* < 0.001, *η*^2^ = 0.58; the cheating rate in the score condition (*M* = 0.44, SD = 0.04) was significantly higher than in the money condition (*M* = 0.28, SD = 0.04), *F* (1, 68) = 8.00, *p* = 0.01, *η*^2^ = 0.11. The interaction between the block and condition was not significant, *F* (1, 68) = 0.08, *p* = 0.78 (see Figure 3).

In the score condition, the cheating rate in the first block (*M* = 0.27, SD = 0.04) was significantly lower than in the last block (*M* = 0.63, SD = 0.05), *F* (1, 39) =55.93, *p* < 0.001, *η*^2^ = 0.59 (Table 1). However, there was no significant main effect of reward magnitude level 10 yuan (*M* = 0.42, SD = 0.05) vs. 50 yuan (*M* = 0.48, SD = 0.06), *F* (1, 39) = 0.63, *p* = 0.43, nor was there a significant interaction between block and reward magnitude level, *F*(1,39) = 0.29, *p* = 0.59.

In the money condition, the cheating rate in the first block (*M* = 0.11, *SD* = 0.04) was significantly lower than in the last block (*M* = 0.45, SD = 0.06), *F* (1, 27) = 38.95, *p* < 0.001, *η*^2^ = 0.59 (Table 1). However, neither the main effect of reward magnitude level 10 yuan (*M* = 0.32, SD = 0.06) vs. 50 yuan (*M* = 0.24, SD = 0.06), *F*(1, 27) = 0.91, *p* = 0.35, nor the interaction between block and reward magnitude level, *F*(1, 27) = 1.46, *p* = 0.24, were significant.

## 4 Discussion

The present study embarked on an exploration of how different types of rewards—specifically, score-based versus monetary incentives—and the magnitude of these rewards influence dishonest behavior within a gender judgment task framework. Our initial hypothesis posited that the nature of the reward would significantly impact the propensity of dishonest participants, with the expectation that score conditions would lead to higher proportion of dishonest participants due to the perceived ethical flexibility associated with non-monetary rewards. This hypothesis was grounded in self-concept maintenance theory, which suggests that individuals justify dishonesty to a degree that allows them to maintain a positive self-image. Our findings provide substantial support for this hypothesis, revealing that participants in the score conditions were significantly more likely to engage in dishonest behavior than those in the money conditions.

The significant finding that participants in the score condition demonstrated higher cheating rate than those in the money condition warrants a nuanced interpretation within the context of existing literature on ethical decision-making and the influence of rewards. Our findings are consistent with the recent work by Elaad et al. (2024), who also found that monetary endowments led to more concealment than non-monetary rewards like game points in the Ultimatum Game paradigm. We similarly observed that while the effect was larger for money, cheating still occurred with both reward types. This finding aligns with the theoretical framework of self-concept maintenance theory, suggesting that individuals are more inclined to rationalize dishonest behavior when the rewards are perceived as less tangible or direct (Mazar et al., 2008). The psychological distance provided by score-based rewards, as opposed to the direct financial gain, may facilitate a cognitive separation that enables individuals to maintain their self-concept of honesty while engaging in dishonest acts. This interpretation extends the findings of Ariely and Jones (2012), who demonstrated that the abstraction of rewards (e.g., tokens exchangeable for money) increases the likelihood of cheating by creating a buffer that diminishes the moral weight of the act. The results are also consistent with previous studies (Yechiam and Zeif, 2022, 2023), which found a small positive effect of incentivization on the cognitive reflection test and the conjunction fallacy. The effect was not moderated by the incentive size, implying that it was present for small payoffs as well.

Furthermore, the early initiation of dishonest behavior in the score condition, as compared to the money condition, provides empirical support for the notion that the nature of the reward influences not just the likelihood of dishonesty, but also its temporal dynamics. This finding dovetails with research on the effects of goal setting and achievement on ethical behavior, suggesting that individuals may be quicker to compromise their ethics when they perceive the path to their goal as being less directly tied to monetary gains (Schweitzer et al., 2004). This aspect of our results highlights the importance of understanding how different incentives can trigger unethical behavior at different stages of decision-making processes.

Lastly, the increase in cheating behavior over time, particularly in the score condition, reflects the dynamic nature of ethical decision-making and suggests a process of ethical erosion where initial acts of dishonesty become easier to justify over time. This observation is consistent with the slippery slope model of ethical decision-making, which posits that small unethical acts can gradually lead to larger transgressions as the individual’s moral thresholds are lowered (Welsh and Ordóñez, 2014). Our findings extend this model by illustrating how the type of reward can influence the rate at which this ethical erosion occurs, with intangible rewards accelerating the process.

Moreover, our study’s observation that the magnitude of rewards did not significantly affect the cheating rate or the timing of initial cheating contrasts with some expectations based on traditional economic models, which would predict an increase in dishonesty with higher rewards. This finding contributes to a growing body of literature questioning the linear relationship between incentive size and unethical behavior, suggesting that factors such as reward type and the opportunity to rationalize one’s actions may play more critical roles (Gneezy, 2005; Shalvi et al., 2011). It underscores the complexity of human motivation and ethical decision-making, indicating that the intrinsic versus extrinsic nature of rewards and their perceived ethical implications can override the simple calculus of risk and reward. This discrepancy could be attributed to the specific context and nature of our experimental task, which may not directly equate to the real-world scenarios where larger financial stakes are involved. Our findings imply that once the decision to engage in dishonest behavior is rationalized, the magnitude of the reward becomes a secondary consideration, underscoring the importance of psychological factors over purely economic ones.

One notable limitation of our study pertains to the experimental design, wherein the possibility of cheating by participants was inherently conspicuous, making it relatively straightforward to identify dishonest behavior. This aspect of the paradigm implies that the cheating observed within our study occurs under conditions where detection is almost guaranteed, potentially influencing the nature and dynamics of the dishonest behaviors we recorded. Such a design contrasts with real-world scenarios where individuals might engage in dishonest acts under the belief or hope that their actions will go undetected, or where the probability of being caught is perceived as low. Therefore, it is crucial for future research to consider experimental designs that more closely mimic the complexity and subtlety of real-world cheating scenarios. This might involve creating conditions where the likelihood of detection is variable or less certain, thereby providing a richer context for exploring how individuals navigate the ethical dilemmas associated with different forms of incentives.

## 5 Conclusion

The findings reveal that the nature of rewards significantly affects the propensity of dishonest participants, indicating that intangible rewards facilitating rationalizations for dishonesty more readily than tangible financial incentives. This pattern persisted irrespective of the magnitude of the rewards, suggesting that the type of incentive plays a more critical role than its size in influencing unethical behavior. These results support self-concept maintenance theory, highlighting the importance of psychological distance and the mechanisms of rationalization in ethical decision-making.

## Data availability statement

The raw data supporting the conclusions of this article will be made available by the authors, without undue reservation.

## Ethics statement

The studies involving humans were approved by Ethics Committee of Ludong University. The studies were conducted in accordance with the local legislation and institutional requirements. The participants provided their written informed consent to participate in this study.

## Author contributions

G-ZC: Writing – review & editing. F-FZ: Formal analysis, Writing – original draft. H-ML: Conceptualization, Writing – original draft. Y-WW: Investigation, Supervision, Writing – original draft. W-JY: Validation, Writing – original draft.
